# The effects of temperature changes on *Totoaba macdonaldi* larval development, growth, and respiratory rates

**DOI:** 10.1007/s10695-025-01595-8

**Published:** 2025-10-22

**Authors:** Á. H. Hernández-Montiel, E. Larios-Soriano, S. Sánchez-Serrano, E. Gisbert, C. True, L. M. López, M. A. Galaviz-Espinoza

**Affiliations:** 1 Facultad de Ciencias Marinas, Universidad Autónoma de Baja California (UABC), Carretera Transpeninsular Ensenada - Tijuana No. 3917, Col. Playitas, 22860 Ensenada, B.C., Mexico; 2 IRTA, Centre de Sant Carles de La Ràpita, Aquaculture Program, Crta. Poble Nou Km 5.5, 43540 Sant Carles de La Ràpita, Spain

**Keywords:** Totoaba, Larvae, Temperature, Thermal Stress, Growth, Development

## Abstract

Understanding the effects of environmental temperature on marine fish larvae is crucial for optimizing feeding regimes and promoting optimal development and growth from a zootechnical perspective in aquaculture practices. The present study investigated the impact of different rearing temperatures on development, growth, and oxygen consumption rates of *Totoaba macdonaldi* larvae. An open-flow seawater recirculating system was used in which larvae (150 specimens/L) were exposed in triplicate to four rearing temperatures (20, 24, 26, and 28 °C) for 24 days under standard larval rearing conditions. Larvae were sampled at 7, 12, 16, 20, and 24 days post hatching (DPH) to assess growth performance, survival, histological condition, and respirometry. At the end of the trial, the highest total length (TL) and body weight (BW) values were observed at 26 °C, with larvae displaying negative allometric growth in terms of TL and BW, similar to those reared at 24 and 28 °C. In contrast, larvae reared at 20 °C showed lower growth and an isometric growth pattern. No significant differences in Fulton’s condition factor (K) were detected among treatments. Survival rates were the highest at 28 °C, while increased cannibalism at 26 °C was associated with elevated specific growth rates and thermal growth coefficients. Histological analysis revealed better structural organization in the gills and liver of larvae reared at 26 °C, whereas other treatments showed signs of inflammation. Respirometry results indicated that larvae reared at 20 °C had significantly higher metabolic rates. However, these differences diminished by DPH 20 and 24, likely reflecting developmental changes and phenotypic plasticity during the larval-to-juvenile transition. Based on physiological and metabolic indicators, a rearing temperature of 26 °C is recommended for *T. macdonaldi* larviculture.

**Supplementary Information:**

The online version contains supplementary material available at 10.1007/s10695-025-01595-8.

## Introduction

Totoaba (*Totoaba macdonaldi*) is a carnivorous marine fish species with indirect ontogenetic development characterized by progressive physiological adaptations to biotic and abiotic factors (Copp and Kováč 1996). For example, early development of the gill respiratory system supports and eventually replaces gas exchange through the skin during larval development, shifting from passive skin to active branchial respiration (Rombough 1999). This respiratory transition enhances aerobic capacity, increasing energy availability for feeding and evading predators during the larval-to-juvenile transition. Moreover, the increase in energy supply and demand is associated with anaerobic metabolism development and acid digestion, which, may be associated with the transition from larval to a juvenile phenotype (Galaviz et al. 2012; Xue et al. 2012; Srichanun et al. 2013; Canada et al. 2017; Khoa et al. 2019; Gamberoni et al. 2021; Larios-Soriano et al. 2023).

Totoaba as a poikilothermic organism, environmental temperature affects fish physiology by modifying metabolic rates and energy homeostasis, which directly affects the organism’s development. These effects have been associated with changes in water viscosity, ion concentration and gas solubility due to water temperature variations (Volkoff and Rønnestad 2020). Marine fish raised at suboptimal temperatures struggle to regulate body temperature due to their poikilothermal condition, which maintains metabolic and energy processes (Pepe-Victoriano et al. 2021; Roman et al. 2019). For example, in a confined environment under low temperatures, a reduction in feed intake was observed, resulting in slower growth rates and immunological and metabolic disorders (Richard et al. 2016). Conversely, increasing temperature inversely affects concentration and dissolved oxygen availability, resulting in a reduced somatic growth as observed in some fish species and other poikilothermic organisms (Burke et al. 2022; Lavin et al. 2022). Furthermore, high rearing temperatures increase oxidative stress damage, decrease mitochondrial respiratory activity, and compromise the overall fitness of the organism (Keil et al. 2015). Thus, temperature is considered the most important abiotic factor that modulates development and growth throughout the life cycle of poikilothermic organisms (Réalis-Doyelle et al. 2016).

In aquaculture, suboptimal water temperatures may prolong larval rearing periods, thereby increasing production costs associated to infrastructure use, manpower, and feeding. These conditions may also lead to malnutrition caused by decreasing appetite, reducing feed conversion, and ultimately, limiting available energy that larvae need for proper growth, morphogenesis, and development (Lema et al. 2019; Dahlke et al. 2020). As previously mentioned, a decrease in skin respiration—coupled with an increase in gill gas exchange—is observed during the transition from larval-to-juvenile stages (Kupren et al. 2014). This shift in respiratory mechanism improves the efficiency in CO_2_ and O_2_ exchange, which could enhance energy metabolism, increase available energy for movement, promoting foraging behaviour (Luiza et al. 2020). This situation is translated into body allometric changes in the head region and tail, which are linked to critical ontogenetic development milestones, such as the that of the gill apparatus, as well as the notochord and caudal fin development (Gibb et al. 2006; Hu et al. 2018; Downie et al. 2020). Higher temperatures have been observed to accelerate growth and development, but the thermal range that promotes totoaba growth without compromising physiological and metabolic performances is still unknown. Furthermore, temperature-induced somatic growth rate acceleration may lead to adverse outcomes, such as increased cannibalism or misalignment between rearing practices and larval morphogenesis. In summary, suboptimal temperatures, combined with changes in body mass, activity levels, and feeding result in morphological changes, which in turn cause physiological and energy demand alterations to maintain metabolic rates leading to various developmental issues (Steffensen 2005). Evaluating fish larval morphological, physiological, and metabolic responses at different temperatures is essential to identify energy expenditure demands, which could allow a feeding protocol development designed to meet energy requirements at different environmental temperatures, improving larval physiological performance.

Farming *Tototaba macdonaldi* began at UABC (Universidad Autónoma de Baja California) in 1993 for conservation and restocking purposes (True et al. 1997). According to the IUCN, *T. macdonaldi* conservation status is listed as vulnerable, primarily due to intense and unregulated fishing pressure on wild populations driven by the high value of the species’ swim bladder in the Asian market. Today, the species is cultured in various breeding centers and commercial farms, making it an important commercial aquaculture product for Northwestern Mexico (López et al. 2015; Maldonado-Othón et al. 2022). However, high mortality rates during larval stage remain a challenge probably associated with biotic and abiotic factors, such as weaning practices and/or inadequate maintenance of physicochemical water quality parameters (Mata-Sotres et al. 2015; García-Ortega and Lazo 2004; Giffard-Mena et al. 2020). The present study hypothesizes that establishing optimal or near-optimal water temperature regime for *T. macdonaldi* larvae may improve current rearing practices. The main objective is to evaluate the effect of different rearing temperatures (20, 24, 26, and 28 °C) on transition from larval-to-juvenile stages in *T. macdonaldi*. Therefore, water temperature for totoaba larval rearing should be determined by comparing biological and physiological variables, such as survival, somatic specific growth rates, Fulton’s condition factor, and changes in the histological organization in gills and liver, as well as in their respiratory metabolism. Considering the vulnerable conservation status of this species, the significance of the present study goes beyond improving aquaculture production techniques, since by focusing on a species of conservation concern, the present research contributes valuable knowledge that may support both sustainable aquaculture practices and conservation efforts for a threatened species.

## Materials and methods

### Larval rearing and sampling

Fertilized totoaba eggs were obtained from a captive broodstock kept in two separate groups (30 fish per group; 25–30 kg in weight, sex ratio male to female of 2:1) held at the Faculty of Marine Sciences at UBP (Unidad de Biotecnología en Piscicultura) at UABC (Universidad Autónoma de Baja California), Mexico. Gonadal maturation in adult totoaba was induced using photothermal control to mimic natural seasonal cycles. Ovulation and spermiation were stimulated using [des-Gly10, D-Ala6]-LHRH ethylamine acetate salt hydrate (SIGMA^R^). Fertilized, buoyant eggs were collected 28–36 h after spawning, treated with 100 mg L^−1^ formalin for 1 h, rinsed and stocked at a density of 100 eggs L^−1^ in 2200-L cone bottom tanks with 24 °C seawater recirculated at 1.5–2.0 L min^−1^ through a fluidized bed biofilter, ultraviolet sterilizer and foam fractionator. Eggs hatched approximately 20 h after incubation. Egg viability was estimated by quantifying several 1-ml samples under a stereoscopic microscope as described by Kjørsvik et al. (1990).

After hatching, totoaba larvae were stocked at a density of 150 individuals L^−1^ in a system consisting of 12 conical fibreglass tanks, each with a 100-L water capacity. The tanks were connected to a recirculating water system equipped with biological and mechanical filters, a ½ horsepower (hp) pump and ultraviolet (UV) sterilization to maintain water quality. Each tank was equipped with a titanium immersion heater connected to a temperature regulation system with digital thermostat (± 0.3 °C accuracy). Larval rearing water temperatures were 20, 24, 26 and 28 °C, with each temperature treatment tested in triplicate. Feeding protocols followed those described by Galaviz et al. (2015) and were performed three times daily (08:00, 12:00, and 18:00 h). Exogenous feeding began once the yolk sac and oil droplet were fully consumed, which occurred between 3 and 4 days post hatching (DPH). During larval rearing, water flow increased to 4-L min^−1^ when feeding began with rotifers (*Brachionus plicatilis*) enriched with a commercial emulsion (Selco-S.presso-INVE Aquaculture, Belgium) and *Artemia* metanauplii. The feeding schema consisted of larvae with enriched rotifers from 4 to 12 DPH, using a combination of enriched rotifers and *Artemia* metanauplii from 10 to 16 DPH, whereas larvae from 16 to 24 DPH were co-fed with *Artemia* metanauplii and a micro-diet (Otohime B 250–360 mm, Japan); proximate composition: protein 52.1%, lipid 16.3%, ash 11.2%). For sampling purposes, larvae (*n* = 15) from each tank were euthanized by placing them in water saturated with 1:1 ethanol and 70% of eugenol (E51791, Sigma-Aldrich, USA) and then rinsed with distilled water for further analyses. Larval samples were collected at 7, 12, 16, and 20 DPH and juveniles at 24 DPH.

### Survival and growth measurements

Survival (S) was calculated at the end of the experimental study for each of the experimental tanks by averaging over the three replicates of each treatment according to the following equation:

$$\text{S}\,\left(\text{\%}\right)=\left(\text{Nf}/\text{Ni}\right)\times 100, \text{where Nf and Ni are the final and initial number of fish},\text{ respectively}$$

At the end of the trial, the percentage of early juvenile cases with severe lordosis was calculated according to the following formula:

$$\text{sl}\,\left(\%\right)=\left(\text{fl}/\text{N}\right)\times 100$$

where fl was the number of fish with lordosis and *N* the total number of fish examined.

For each sampling day (7, 12, 16, 20, and 24 DPH), larvae were collected from each tank to measure total length (snout tip to longest caudal lobe) and wet weight. A total of 15 larvae per tank were sampled at 08:00 h under 14 h fasting conditions. Larvae were weighed using a 0.0001 g (± 0.0001 g) precision balance (Sartorius AG CP224S, Germany) and measured for body length under a microscope (MEIJI EMZ-13TRH, Japan), which were superimposed on a Neubauer camera. Images were captured using ImageJ v1.53j software, which was used to measure the exact length of each larva to calculate the following parameters:

Specific growth rate in length (SGR % day^−1^): SGR = 100 (ln TLf − ln TLi)/t, where TLf is the final total length (mm), TLi is the total initial length (mm) and *t* is the time in days.

Allometric growth (body weight in relation to larval size in length): BW = aL^b^, where BW is the body weight (g), *L* is the total length (cm), *a* is the intercept of the linear regression, and *b* is the slope of the line.

To determine the type of allometric growth: log (Wti) = log (a) + blog (Lti) + Ei, where log (Wti) = *y*, *b* is the slope, log (a) is the intercept, and Ei is the multiplied error of the i-th fish.

With this regression growth type was assessed: isometric when H0: *b* = 3, while H1: *b* ≠ 3 indicates allometric growth. The maximum length of the lowest growing treatments was then compared to the highest growing treatment using linear regression with log-transformed length data.

Fulton condition factor (*K*) was obtained using the formula: *K* = 100 × (BW/TL^3^), where BW is the wet body weight (g) and TL is the total length (cm).

The thermal growth coefficient (TGC) was calculated as follows: TGC = [(3√BWt—3√BWo)/(T × t)] × 1000, where BWt is the final BW, BWo is the initial BW, *T* is the average temperature for the time interval considered, and *t* is the time.

### Histological analyses

Larvae were collected at 7, 12, 16, 20, and 24 DPH for histological analyses (*n* = 20 per day from each tank). After being euthanized as previously described, larvae were fixed in 4% paraformaldehyde for 12 h; then, they were dehydrated in an increasing series of graded ethanol in a Leica tissue processor (model TP1020, Leica Microsystems, USA) and embedded in paraffin. After that, 4–5-µm-thick longitudinal sections were made with a Leica manual rotary microtome (model RM2125RTS, Leica Microsystems, USA) and stained with Alcian Blue and Mayer’s hematoxylin (Merck, MA, USA) to observe acidic mucins and mucopolysaccharides. A cell morphometric analysis was performed to evaluate differences between cells (hepatocytes and gill cells) from the different treatments; cell and nucleus diameter measurements were taken from 30 randomly selected organisms for thermal treatment to calculate cytoplasm diameter using ImageJ v1.53j software (Schneider et al. 2012). While for the gills, the width of primary lamellae (PL) and secondary lamellae (SL) was measured in 30 randomly selected organisms.

### Metabolic rate determination

Oxygen consumption rate was determined by the difference between initial and final oxygen of the water contained in the respirometry chamber. This system consists of a 24-well glass plate each one with a capacity of 750 μL. A Dish Reader® oxygen sensor (PreSens, Regensburg, Germany) is below each of the wells with an accuracy of ± 1% O_2_. Dissolved oxygen was measured in triplicate, placing larvae from each experimental condition (20, 24, 26, and 28 °C) at different post hatching ages [7 DPH (*N* = 10), 16 DPH (*N* = 5) and 24 DPH (*N* = 1)] following the protocol used by Larios et al. (2023). Oxygen consumption data were expressed as mg O_2_ larva^−1^ h^−1^. Temperature quotient (Q_10_) was evaluated following the equation of Schmidt-Nielsen (1997): *Q*_10_ = (TCO2/TCO1) ^(10/T2−T1)^, where *T* is the water temperature, TCO1 is the initial oxygen consumption rate, and TCO2 is the final oxygen consumption rate.

### Statistical analyses

The analyses were performed using the mean and standard deviation; normality and homogeneity of variances were evaluated using Shapiro–Wilk’s and Levene’s tests, respectively. To compare final length and weight, survival, severe lordosis, a thermal growth coefficient and one-way analysis of variance (ANOVA) were conducted, followed by Tukey’s multiple comparison of means test at 95% confidence level. Additionally, normality and homogeneity of variances were evaluated to apply a mixed-effects ANOVA, for which sphericity was assessed using the Mauchly test; the Greenhouse–Geisser correction was applied when the covariance between matrices was not met for O_2_ consumption for DPH 7, 16, and 24 and hepatocyte cytoplasm diameter between DPH 16 and 24. A post hoc pairwise *t*-test was then conducted, considering asymmetry of the model. Subsequently, variables that did not meet normality and homogeneity of variance criteria, such as SGR, K, primary lamella (PL), and secondary lamella (SL), were compared by assessing the sum of ranks for each group with Kruskal–Wallis’ test; Dunn’s post hoc test followed with *P* value adjustment by Bonferroni method (rstatix v0.7.0), using R version 4.2.1 and Rstudio version 2022.07.0 + 548. A Spearman correlation (stats v4.2.1) was also performed between O_2_ consumption, feed type, and growth parameters: age in DPH, L, W, SGR, and K condition was visualized with the corrplot package v0.92, eliminating the values of variables with no significant correlation.

A least square adjusted local regression model with confidence intervals of 0.95 was performed to compare length (cm) and weight (g) at different temperatures, with span = 0.75 (stats v4.2.1) (Cleveland et al. 2017), (ggplot2 v3.3.6). A linear regression was performed with the logarithm of length (log L) and weight (log W) to assess allometric growth at different temperatures (FSA v0.9.3). Another linear regression followed with DPH and log TL to compare growth time of the different temperatures with one with the highest growth. To reduce bias in weight predictions relative to length, the log-transformed allometric values were corrected by multiplying them by a logarithmic correction factor (Sprugel 1983). Finally, a principal component analysis (PCA) was performed to determine each variable contribution per component about experimental larval rearing conditions; then, clusters were made to assess whether significant differences existed between the variables with the highest eigenvalue.

## Results

Survival rates at 28 °C (23.05 ± 1.06%), followed by 26 °C (20.83 ± 0.75%) and 24 °C (18 ± 3.47%) showed no statistically significant differences (*p* > 0.05). However, significantly lower survival (*p* < 0.05) was observed from the 20 °C treatment (10.4 ± 2.5%) to the remaining temperature groups. Regarding severe lordosis, a higher number of cases were identified at 20 °C (40.0 ± 4.3%), followed by 24 °C (33.33 ± 3.0%), 28 °C (15.3 ± 4.33%), and 26 °C (11.56 ± 6.23%), where significant differences were observed from 20 to 24 °C treatments with 26 and 28 °C (*p* < 0.05). A negative correlation was observed between survival rates and vertebral column deformities of − 62% (*p* < 0.05). While with respect to temperature they showed a correlation of 78% and − 87%, respectively. In addition, only the 26 °C treatment was identified with thermal growth coefficient (TGC), showing significant differences with the rest of the groups (*p* < 0.05).

**Fig. 1:**
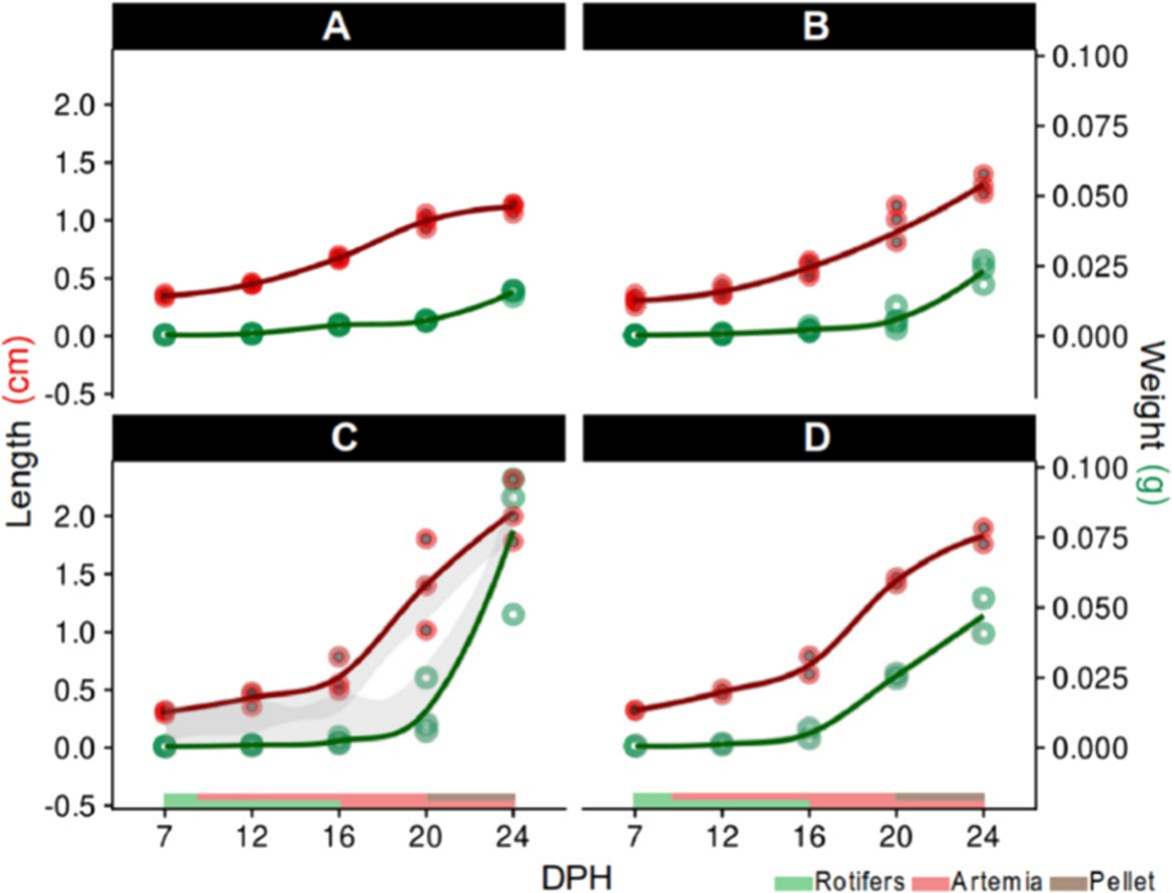
Growth at different temperatures in totoaba (*Totoaba macdonaldi)* larvae. **A–D** Local regression models with 95% confidence intervals (non-normalized data), to visualize the correlation among feed type, length, and weight at 20 °C (**A**), 24 °C (**B**), 26 °C (**C**), and 28 °C (**D**), respectively

As expected, water temperature influenced larval growth performance under current experimental conditions (Fig. 1). By the end of the trial, the highest BW and TL average were observed in totoaba larvae reared at 26 °C (BW = 0.078 ± 0.02 g and TL = 2.03 ± 0.27 cm) (Fig. 1C), followed by those reared at 28 °C (BW = 0.047 ± 0.006 g and TL = 1.83 ± 0.07 cm) (Fig. 1D), 24 °C (BW = 0.023 ± 0.004 g and TL = 1.31 ± 0.08 cm) (Fig. 1B), and 20 °C (BW = 0.016 ± 0.001 g and TL = 1.11 ± 0.04 cm) (Fig. 1A). In contrast, the 26 and 28 °C treatments presented significant differences with regard to the 20 and 24 °C treatments in TL (ANOVA, F (3,8) = 38.62, *p* < 0.05) and BW (ANOVA, F (3,8) = 27.83, *p* < 0.05).

On the other hand, the linear regression model between BW and TL (Fig. S1) showed similar variability patterns among water temperature treatments, arranged from 28 °C (*R*^2^ = 96.17%), followed by 24 °C (*R*^2^ = 95.92%), 26 °C (*R*^2^ 95.54%), and 20 °C (*R*^2^ = 94.32%) based on *R*^2^ values. Subsequently, the evaluation of the null hypothesis to determine growth patterns, whether isometric (H₀: *β* = 3) or allometric (H₁: *β* ≠ 3), indicated that larvae reared at 20 °C showed isometric growth (*β* = 2.75 ± 0.16), as the 95% confidence interval for the slope (CI₉₅%) ranged from 2.41 to 3.08, encompassing the value of 3. In contrast, larvae from the other temperature treatments showed slightly negative allometric growth. The degree of negative allometry increased with temperature: 24 °C (*β* = 2.68 ± 0.12, CI₉₅% = 2.43–2.94), 26 °C (*β* = 2.51 ± 0.14, CI₉₅% = 2.20–2.82), and 28 °C (*β* = 2.49 ± 0.16, CI₉₅% = 2.11–2.88).

**Fig. 2:**
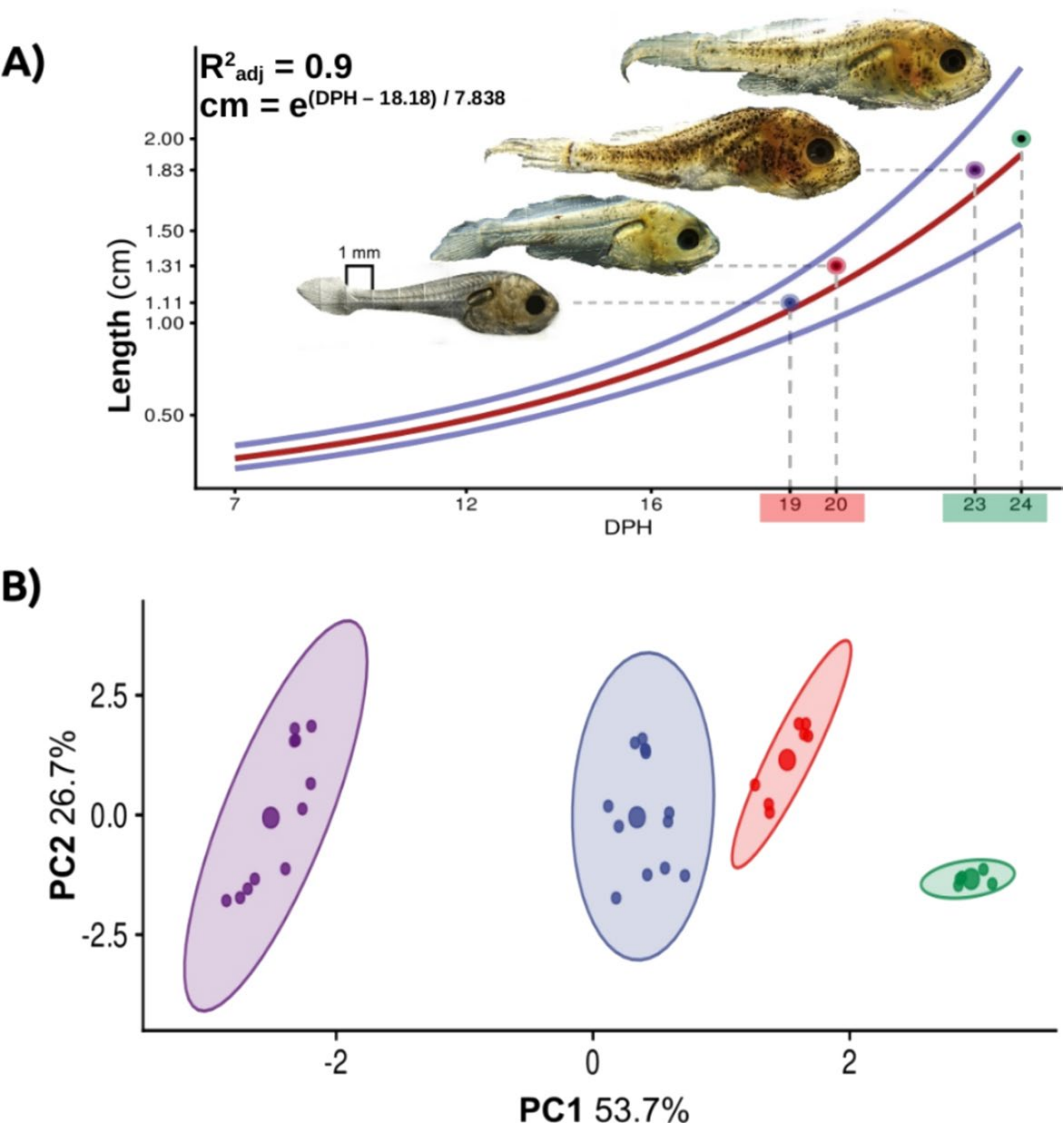
Delay in final length relative to temperature and grouping of factors involved in totoaba (*Totoaba macdonaldi*) larval growth. **A** Growth curve for treatment at 26 °C, where blue, red, purple, and green dots indicate the final average size for treatment at 20, 24, 28, and 26 °C. Larval images are 24 days post hatching (DPH) (blue lines represent confidence intervals). The dotted lines indicate the DPH where each treatment reached its maximum size, with respect to 26 °C at 24 DPH variance. **B** Grouping the data in four clusters (k), where k1 (purple, *n* = 12; DPH 7, Tm: all), k2 (blue, *n* = 12; DPH 16, Tm: all), and k3 (red, *n* = 7; DPH 24, Tm: 20, 24 °C) are related to PC1 and k4 (green, *n* = 5; DPH 24, Tm: 26, 28 °C) to PC2

To compare the final TL between treatments, a growth curve of larvae exposed to 26 °C was used as a reference (Fig. 2). In this curve, the DPH were identified where the TL in each treatment (20, 24, and 28 °C) was the same TL value reached at 24 DPH in the 26 °C treatment. The results showed that the final TL of totoaba larvae when reared at 28, 24, and 20 °C was found at 23, 20, and 19 DPH when compared to the reference standard of 26 °C, respectively (Fig. 2A), which indicated that rearing totoaba larvae at 26 °C reduced larval rearing time by 1, 4, and 5 days, respectively. Furthermore, the PCA analysis revealed that 80.4% of variance was explained by the PC1 (DPH, feed type, and oxygen consumption) and the PC2 (SGR and Tm). Four K-mean clusters on PCA (k) were identified, which discriminated among experimental water temperatures; in particular, the four cluster k4 (*n* = 5) was composed of larvae aged 24 DPH from the 26 and 28 °C treatments, while k3 (*n* = 14) was composed of larvae aged 24 DPH from the 20 and 24 °C groups. The variation of k4 was observed mostly explained by PC1 and k3 by PC2 (Fig. 2B).

The SGR value for larvae reared at 26 °C (11.05 ± 0.43% TL day^−1^) was similar to that of congeners kept at 28 °C (10.17 ± 0.18% TL day^−1^) (*p* > 0.05). However, significant differences were observed with larvae reared at 20 °C (6.9 ± 0.35% TL day^−1^) and 24 °C (7.79 ± 1.65% TL day^−1^) (Fig. S2a, *p* < 0.05). In addition, *K* did not show significant differences between treatments (Fig. S2b, *p* > 0.05). However, factor values were negatively correlated with O_2_ consumption rates (− 45%), age in DPH (− 44%), TL (− 54%), and feed (− 44%) (Fig. S2c). This pattern aligns with the observed positive correlation of SGR in length with temperature (82%) and, to a lesser extent, with BW (14%). These findings are consistent with the previously described negative allometric growth in BW relative to TL in larvae reared at 24, 26, and 28 °C.

**Fig. 3:**
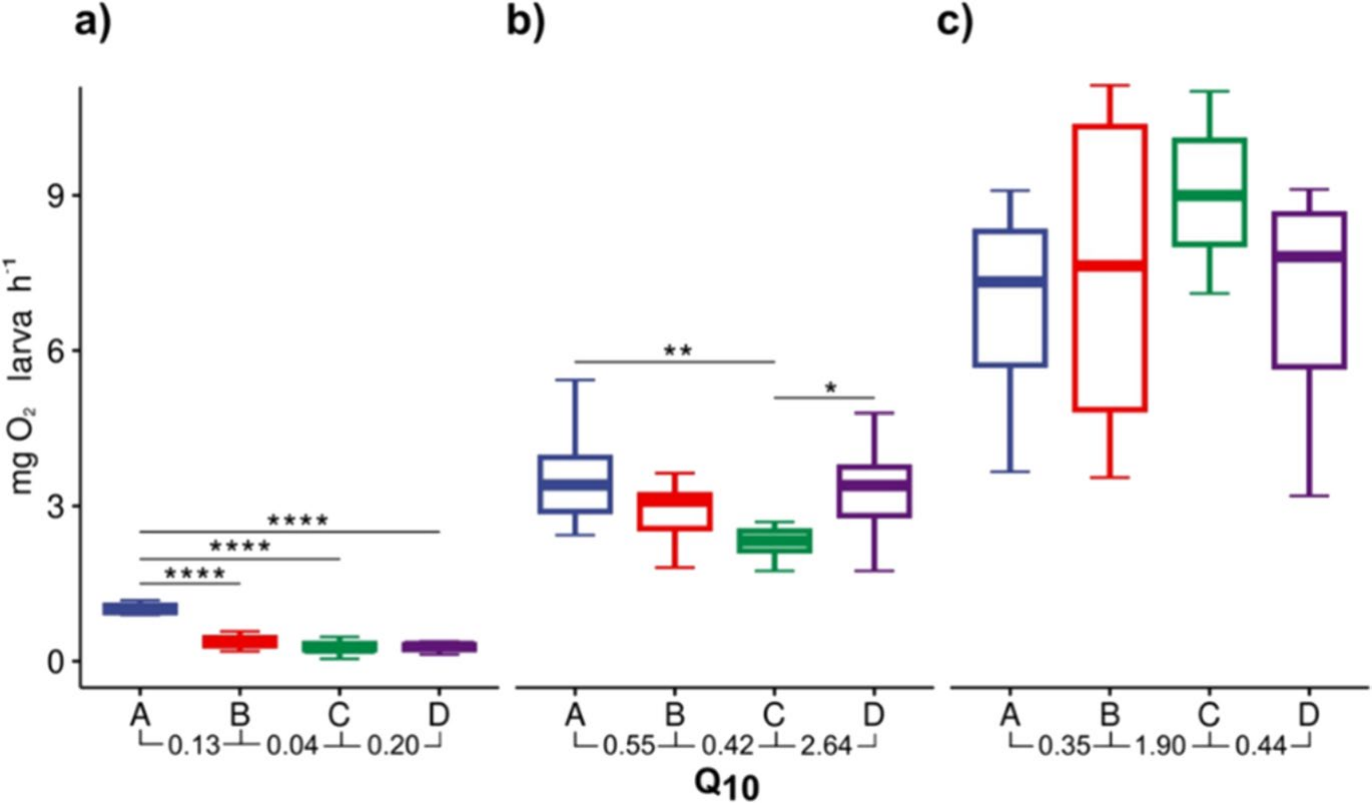
Oxygen consumption (mg O_2_/larvae/h^−1^) of totoaba (*Totoaba macdonaldi*) larvae exposed to different temperatures for 24 days post hatching (DPH). **a** Post hoc analysis for DPH 7 with significant differences (*p*. adj < 0.05) between A–B, A–C, and A–D. **b** Post hoc analysis for DPH 16 with significant differences (*p* adj < 0.05) between A–C and C–D. **c** Post hoc analysis with not significant differences (A = 20 °C, B = 24 °C, C = 26 °C, and D = 28 °C). Mixed-effects ANOVA and Dunn’s post hoc test with Bonferroni adjustment between 7, 16, and 24 DPH, where significant differences were observed (ANOVA, F (1.39, 12.48) = 5.42, *p* < 0.05, *η*2 = 0.35) with a greater effect size of DPH on oxygen consumption (*η*2 = 0.94). While by treatment, C had the largest effect size (*p*. adj < 0.05, *η*2 = 0.96)

For oxygen consumption rates, the post hoc analysis showed significant differences (*p* < 0.05) between average oxygen consumption rates in 7 DPH larvae reared at 20 °C (0.97 ± 0.15 mg O_2_ larva^−1^ h^−1^) with regard to larvae kept at 24, 26, and 28 °C (0.82 ± 1.04, 0.27 ± 0.10 and 0.26 ± 0.08 mg O_2_ larva^−1^ h^−1^, respectively). In this case, the lowest *Q*_10_ value was observed in the range from 24 to 26 °C (Fig. 3a). At 16 DPH, significant differences in oxygen consumption were observed from 20 °C (3.48 ± 0.81 mg O_2_ larva^−1^ h^−1^) to 26 °C (2.30 ± 0.45 mg O_2_ larva^−1^ h^−1^), as well as with 26 °C with 28 °C (3.37 ± 1.03 mg O_2_ larva^−1^ h^−1^). Additionally, the lowest *Q*₁₀ value at this age was recorded from 24 to 26 °C (Fig. 3b). On the other hand, at 24 DPH, no significant differences were observed in oxygen consumption among treatments (*p* > 0.05), with a higher numerical *Q*_10_ value found in the range from 24 to 26 °C (Fig. 3c). Oxygen consumption was statistically analysed using a mixed-effects ANOVA where significant differences (*p* < 0.05) and a larger effect size were observed for the variable age (DPH; *η*^2^ = 0.93) compared to water temperature (*η*^2^ = 0.58). The experimental group with the largest effect size during development was 26 °C (*η*^2^ = 0.96), followed by 20 °C (*η*^2^ = 0.80), 28 °C (*η*^2^ = 0.80), and 24 °C (*η*^2^ = 0.76).

**Fig. 4:**
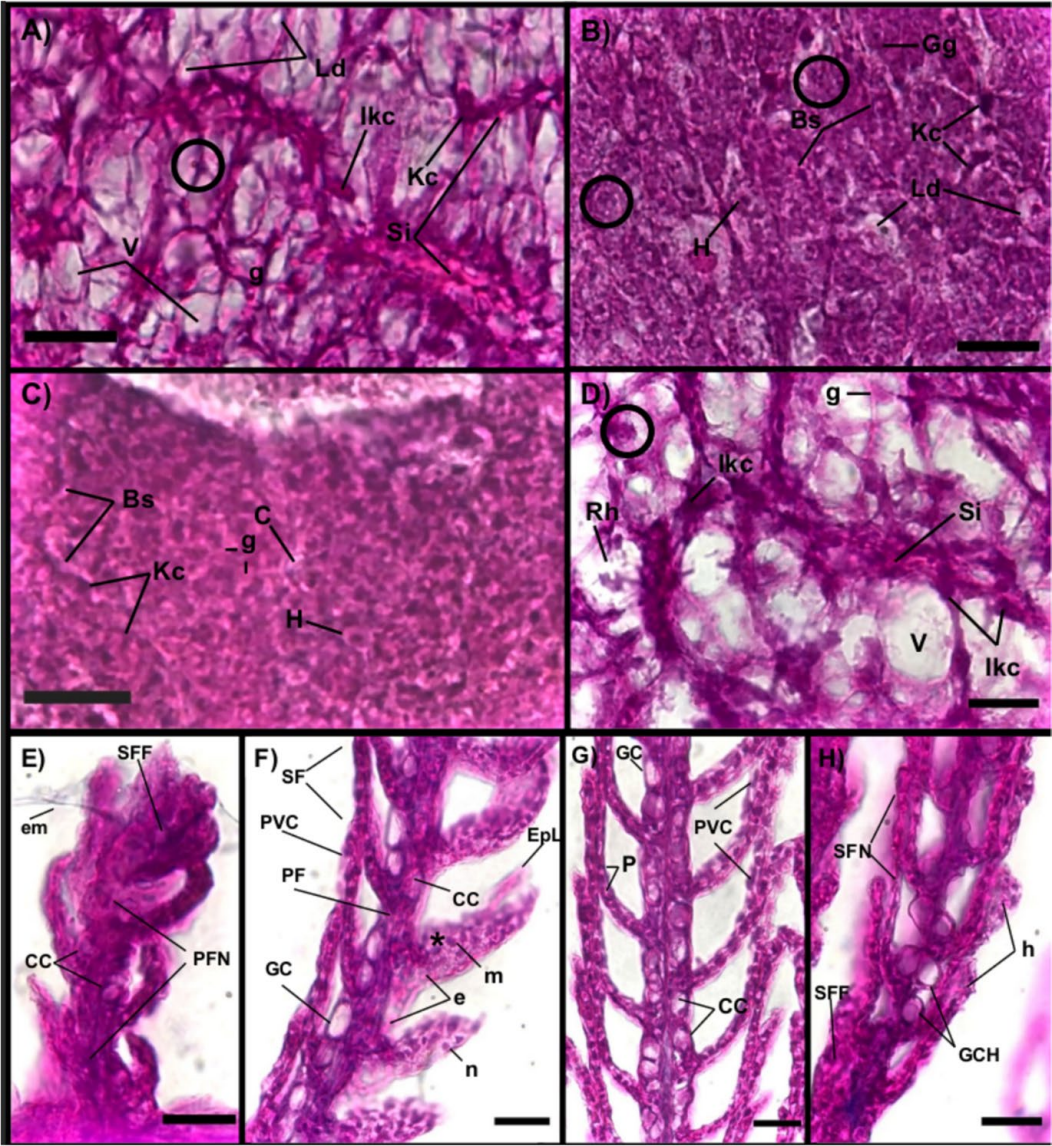
Histology of liver and gills of totoaba (*Totoaba macdonaldi*) larvae at 24 days after hatching (DPH). **A** and **E** Liver and gill filament of totoaba at 20 °C. **B** and **F** Liver and gill filament at 24 °C. **C** and **G** Liver and gill filament of totoaba at 26 °C. **D** and **H** Liver and gill filament at 28 °C. AB staining; magnification bar = 20 μm. Abbreviations: PVC, pavement cells; h, hyperplasia; GCH, goblet cell hypertrophy; PF, primary filament; SF, secondary filament; e, erythrocytes. *: lamellae congestion: em, excess of mucus secretion; P, pillar cells; SFF, secondary filament fusion; EpL, cell detachment; PFN, primary filament necrosis; CC, chloride cells; Bs, blood sinusoid. Circulus: pyknotic nucleus; V, vacuolization; Si, inflamed sinusoid; Kc, Kupffer cells; IKC, increased Kupffer cells; Rh, hepatocyte rupture; H, hepatocyte; C, central vein; Ld, lipid droplets; g, glycogen; Nc, nucleus compression; Cs, sinusoidal congestion

The histological organization of liver and gills was analysed at 16 and 24 DPH. Regarding the histological organization of the liver in larvae aged 16 DPH, a smaller diameter of hepatocytes was observed in larvae reared at 20 °C (3.47 ± 0.89 μm) when compared to their congeners reared at 28 °C (4.22 ± 1.08 um), 26 °C (5.47 ± 1.78 μm), and 24 °C (7.58 ± 1.95 μm) (Fig. S3). At 24 DPH, the smallest hepatocyte diameters were observed in larvae kept at 26 °C (3.22 ± 0.83 μm), followed by those reared at 24 °C (4.06 ± 1.36 μm), 20 °C (8.80 ± 3.17 μm), and 28 °C (12.97 ± 4.75 μm) (Fig. 4A–D). The mixed-effects ANOVA used to assess the hepatocyte cytoplasm diameter from 16 to 24 DPH showed a larger effect of water temperature (*η*^2^ = 0.332) than age in DPH (*η*^2^ = 0.159). In particular, 28 °C presented a larger effect size (*η*^2^ = 0.825), followed by 24 °C (*η*^2^ = 0.691), 20 °C (*η*^2^ = 0.677), and 26 °C (*η*^2^ = 0.441). The post hoc analysis at the age of 16 DPH (Fig. S4a) showed a smaller cytoplasmatic diameter with significant differences (*p* < 0.05) between larvae reared at 20 °C compared to those reared at 24, 26, and 28 °C, while at 24 DPH (Fig. S4b), significant differences were observed between 20 and 28 °C compared to 24 °C and 26 °C (*p* < 0.05).

Furthermore, variation in oxygen consumption observed in larvae aged 24 DPH at 24 °C might be related to the physical integrity of the gills with a negative correlation (− 60%) between secondary lamella (SL) width and oxygen consumption (*p* < 0.05), where treatment at 24 °C (12.45 ± 4.32 μm) differed from that at 20 °C (7.91 ± 1.03 μm), 26 °C (7.43 ± 1.68 μm), and 28 °C (8.32 ± 1.67 μm) (*p* < 0.05). On the other hand, a positive correlation (45%) was observed between primary lamella (PL) and K (*p* < 0.05) with a significantly smaller diameter (*p* < 0.05) between the treatment at 28 °C (10.07 ± 1.92 μm) and 20 °C (18 ± 1.96 μm), 24 °C (19.93 ± 4.18 μm), and 26 °C (19.03 ± 2.89 μm) (Fig. 4E–H).

## Discussion

Larval growth and development are influenced by multiple biotic and abiotic factors; however, in aquaculture where the maximum survival and growth rate are expected, water temperature, monitoring of morphological development, and feed quality and quantity are among the most critical factors (Boglione et al. 2013). Thus, testing these factors in a combined manner was essential for proper tuning up rearing conditions, since correlations exist between growth rate at different temperatures, larval mouth size and prey size available during larval development (Carter et al. 2022). Additionally, the development of key organs involved in primary functions, such as feeding, respiration, and locomotion, is related to allometric growth (Osse and Van der Boogaart 1995), which may be modified by changes in robustness and larval growth rate at different acclimation temperatures or nutrition conditions (Shin et al. 2022; Xu et al. 2023). In this sense, temperature variations affect larval swimming kinetics in rearing tanks and contribute to increased energy expenditure during feeding, which may be also compounded by other suboptimal biotic rearing conditions (Osse and Van der Boogaart 1995). Although totoaba juvenile is considered a thermotolerant fish that exhibits phenotypic plasticity and can compensate for temperature-associated growth variations, thus, modifying fish kinetics (Oufiero and Whitlow 2016; Hernández-Aguilar et al. 2018), prolonged culture periods during larval stages increase costs related to live feed production, maintenance, aeration systems, water heating, manpower, and the risk of exposure to infectious diseases (Araujo et al. 2022). This situation is of special relevance since to our knowledge no studies on larval have been available on development and feeding transition during totoaba weaning; therefore, the present information is of interest for improving larviculture conditions for this fast-growing species.

### Water temperature effect on somatic larval growth

The present study showed that maximum TL (2.03 ± 0.27 mm), SGR and TGC values were observed in totoaba larvae reared at 26 °C. Furthermore, the statistical analysis revealed significant differences in TL and TGC at 26 °C compared to the other rearing temperatures. These results indicated that temperature negatively affected larval growth at 20, 24, and 28 °C compared to 26 °C, showing a quadratic response with regards to larval growth in TL and water temperature. Besides, these results indicated that 26 °C represents an optimal thermal environment for totoaba larvae that promotes nutrient assimilation and weight gain (Jobling 2003). In this case, optimal water temperature ensures the correct water viscosity to support larval survival and development by reducing dragging forces and swimming efforts (Downie et al. 2020). On the contrary, low temperatures increase energy expenditure due to water viscous forces, which delay larval growth, since energy should derive from swimming rather than somatic growth (Osse and Van der Boogaart 2004). On the other hand, temperatures above the optimal range compromise muscle power and feeding ability, as observed in *Sparus aurata*, which showed higher swimming speed when reared at 25 °C and swimming performance declined at 28 °C (Koumoundouros et al. 2009). Previous studies have indicated that 26 °C is the optimal growth temperature for totoaba larvae and juveniles, with an appropriate thermal range from 26.4 to 27.7 °C (Talamas 2001; Yen et al. 2021; Larios-Soriano et al. 2023). The results in the present study are consistent with those observed in other studies indicating that increasing temperature from 24 to 26 °C promoted totoaba somatic larval growth, even though growth rates were compromised at water temperatures lower or higher than this thermal range (i.e. 20 or 28 °C).

It is also relevant to highlight that growth improvements were correlated with increased BW and changes in feed types during weaning at 26 °C. However, larvae exposed at 28 °C showed more elongated bodies due to a pronounced negative allometric growth in terms of BW, whereas larval growth at 20 °C was isometric in both BW and LT (associated with their lower SGR and K condition values). The negative correlation between Fulton’s condition factor, feeding schedule, BW, and TL reinforced the idea that changes in somatic growth were associated with malnutrition during weaning at 20, 24, and 28 °C, which reduced the possibility of reaching the corresponding length and weight to the developmental stage (Osse and Van der Boogaart 2004).

### Survival of totoaba larvae at different farming temperatures

The lowest survival rates were observed at temperatures from 20 to 24 °C, which may be related to metabolic rate change and assimilation efficiency mentioned above. Furthermore, the detection of severe lordosis cases decreased over time, indicating that larvae with deformities did not survive the weaning process. In fish larvae, the vertebral column plays a crucial role in inducing rapid muscle fibre development, which are characterized by strong, short-duration contractions (Blagden et al. 1997). In larvae, the vertebral column and muscle fibre development are essential for foraging behaviour and avoiding predators in the wild or cannibalism in a culture environment. On the other hand, the highest survival rate under current rearing conditions was observed at 28 °C, which was slightly higher than at 26 °C. The general larval condition indicates that cannibalism may have affected the survival rate at 26 °C. Due to the negative larval cannibalism impact on survival rate, the weaning process was anticipated in totoaba at 26 °C to minimize mortality associated with cannibalism (Sánchez-Hernández et al. 2018). To further reduce larval rearing costs, the thermal environment and feeding protocol were identified, optimizing morphological development, energy homeostasis, and growth performance for fish during early life stages. In this context, the cost–benefit of maintaining optimal culture temperatures and implementing effective weaning protocols should be evaluated during larval-juvenile transition. These analyses should include an assessment of the economic profitability index to identify the critical growth point under the most effective feeding rates (Jauralde et al. 2011).

### Water temperature effect on liver histological organization

The liver and its hepatic parenchyma histomorphological organization may serve as a sensitive physiological biomarker caused by nutritionally imbalanced diets, feed deprivation, or energy imbalance caused by rearing conditions (Gisbert et al. 2008). The present study revealed significant differences in the hepatocyte cytoplasm diameter, as well as inflammation signs in the hepatic sinusoids in early juveniles (24 DAH) reared at 20 and 28 °C. The histological alterations found at 20 and 28 °C were not associated with larval growth. Thus, the hypothesis is that alterantions might instead be related to the inflammatory process and vacuolization, respectively, triggered by extreme culture temperatures. In the case of low temperatures, alterations in the production of superoxide radicals, lipid peroxidation levels, and antioxidant enzymes have been observed in totoaba liver (Hernández-Aguilar et al. 2018). On the other hand, an increase in vacuolization was observed in the present study at 28 °C, which may likely be related to a higher micro-diet intake due to an increase in hepatic vacuolization and observed in totoaba larvae from 20 to 28 DPH (reared at 24 °C) during the transition from live prey to the micro-diet and the different quantity and quality of dietary lipids with regards to live prey (Galaviz et al. 2015). Oxidative stress has been observed to negatively affect hepatocyte morphology in cold water fish subjected to a higher (28 °C) culture temperature (Yan et al. 2022; Han et al. 2023). Furthermore, vacuolization, nucleus displacement, and pyknotic nuclei of hybrid catfish hepatocytes increase at high temperatures (Khieokhajonkhet et al. 2022). Heat stress in farmed fish has been associated with increased lipid deposits, and lipid increase in hepatocytes, which may hinder its metabolic performance due to lipid peroxidation and the resulting harmful products that damage cell membranes, leading to adverse morpho-physiological changes that promote cell death (Ayala et al. 2014; Richard et al. 2016). Additionally, lipid peroxidation may affect cell morphology in the liver by disrupting detoxification mechanisms that decrease inflammation (Vinagre et al. 2014; Madeira et al. 2016). For example, reactive oxygen species (ROS) are known to accelerate glycogen-to-glucose conversion, impairing its storage in hepatocytes through oxidative phosphorylation, NADPH oxidase, oxidative bursts of immune cells, and inflammatory responses (Jiang et al. 2017; Ritchie and Friesen 2022; Qin et al. 2023). These findings suggest inflammatory symptoms in the liver of totoaba larvae, possibly linked to the weaning process, micro-diet ingestion, and possible damage due to oxidative stress at these temperatures. During larval transition to juveniles (20 to 28 DPH), the energy metabolism in liver cells may have provided some protection against ROS activity (Häussinger 1998; Tseng and Hwang 2008; Giffard-Mena et al. 2020); however, this transition did not improve liver condition at 20 and 28 °C as heat stress persisted. Therefore, growth results at these temperatures correlate with larval culture conditions that were evident in PC2 of PCA. This thermal stress likely contributed to malnutrition together with premature or delayed feed withdrawal at 20 and 28 °C, respectively. The result suggests that both 20 and 28 °C might represent the thermal limits for larval-juvenile transition in totoaba. Finally, although, no increase in hepatocyte vacuolization was detected at 24 °C in 24 DPH in the present study, longer exposure to formulated feed may be required to observe liver morphological changes at this temperature. Nevertheless, further research is needed to test the former hypotheses correlating oxidative stress with micro-diets and totoaba nutrition.

### Water temperature effect on the gill histological organization

Contrary to that observed in gills of larvae kept at 26 °C, inflammatory processes were present in gills from larvae reared at 20, 24, and 28 °C as indicated by the fusion of secondary filaments and hyperplasia. In addition, the presence of mast cells and neutrophils in secondary filaments was also observed together with an increase in mucous cells at temperatures ranging from 24 to 28 °C. In gills, proinflammatory processes may be indicative of the presence of pathogens, toxicant exposure, and adverse environmental conditions (Pacorig et al. 2022). These proinflammatory processes impair the morphological state of the gills and limits gas exchange, which may compromise larval transition to juvenile stages. In the present study, larvae reared at 24 °C exhibited gills with larger variation in size and width, which negatively correlated with oxygen consumption rates. In addition, significant differences in SL at 24 °C—compared to the other experimental groups—indicated that they were in inadequate rearing conditions (temperature or salinity). In teleost fish, gills function as the main osmoregulatory organ and their euryhaline capacity depends on the number of chloride cells (Samei et al. 2021; Surendran and Ampili 2023). In totoaba larvae cultured at 22 °C, higher survival rates were observed in salinity gradients of 11 to 26 PSU before 20 DPH, while juveniles older than 28 DPH maintained high survival rates in a gradient from 5 to 40 PSU (Giffard-Mena et al. 2020). These results indicate that the transition to juvenile from 20 to 28 DPH may be related to a lower level of oxygen consumption of the larvae at 24 DPH. On the other hand, the morphological state of gills at 26 °C might have contributed to the observed decrease in mortality during the juvenile transition and weaning, whereas the damage observed at PL of 28 °C would indicate proximity to the thermal limit for the condition of gill tissue.

### Water temperature effect on oxygen consumption rates

Determining temperature effect is certainly a challenge on metabolic rate during ontogenetic aquatic organism development. In these studies, in addition to the effect of temperature on metabolic rate, considering different variables is important, such as skin respiration, developing respiratory organs, body, and general physiological state. Nevertheless, interesting tendencies were identified in the present study in *T. macdonaldi*. Firstly, an inversely proportional effect at 7 and 16 DPH on oxygen consumption was observed with respect to the increase in water rearing temperature. In both cases (at 7 and 16 DPH), oxygen consumption rates were higher at a temperature of 20 °C and decreased with increasing temperature values up to 28 °C (at 7 DPH) and 26 °C (at 16 DPH). The same tendency was observed in totoaba larvae exposed for 5 h at different temperatures, higher oxygen consumption related to respiratory metabolism and energy expenditure, and at 21 and 24 °C than 27 °C in larvae aged 6, 8, and 14 DPH (Larios-Soriano et al. 2023). This result might indicate the dependence on oxygen diffusion through the skin in early stages of totoaba development where the gill is not yet complete, and in this particular case, the results might indicate that a delay in gill development could result in increased respiratory effort when totoaba larvae were reared at 20 °C. Since the increase in convective oxygen transport depends on larval body mass (Jacob et al. 2002; Killen et al. 2007), totoaba larvae reared at 20 and 24 °C could be at a disadvantage for both reasons, a reduced growth and poor gill development, which may result in increased stress and high oxygen consumption rated as it was observed in comparation with larvae kept at 26 and 28 °C. Therefore, oxygen consumption through the skin helped larvae to cope with gill damage/developmental delay, which was confirmed by the constant increase in oxygen consumption throughout larval development and transition to the juvenile stage. This result is highly correlated to larval age in DPH (94%), oxygen consumption (93%), and food (94%) with respect to body weight gain.

At 24 DPH, totoaba larvae showed a different trend—a lower oxygen consumption rate at 20 and 24 °C—the highest oxygen consumption rate was observed at 24 °C and a slight reduction at 28 °C. These trends resemble an Arrhenius curve where temperature influences molecule kinetics; the higher the temperature, the higher the oxygen consumption up to a point where the decrease in oxygen consumption is interpreted as loss of aerobic performance or aerobic scope (Clark et al. 2013). At this point in the development of totoaba larvae (24 DPH), evidently, temperature may start to be a determining metabolic rate factor. On the one hand, a value of 1.90 is observed in Q_10_ from 24 to 6 °C, suggesting that the metabolic rate has doubled in this temperature range, reflecting a high susceptibility. Likewise, the highest rate of oxygen consumption was observed at 26 °C, suggesting an aerobic energy activation and greater ATP availability through these metabolic pathways. These results corroborate that the optimal temperature for resting metabolic rate RMR is 26–27 °C.

Finally, larval age had the most evident effect on oxygen consumption rates in older age, indicating higher oxygen consumption rates. This result is consistent with PCA results, where data in PC1 are grouped by DPH and only at 24 DPH is a distinction observed between organisms exposed to 20–24 °C and 26–28 °C, indicating that temperature directly influences larval body size, therefore oxygen consumption. Furthermore, this result also suggests that temperature has a more pronounced effect on growth during the larval stage and transition to juveniles at lower culture temperatures, as indicated by PC2 and the significant correlation between temperature and SGR values. In addition to larval age, oxygen consumption variability was also influenced by temperature and its effects on gill tissue condition and organization. This result is evident from an increase in SL damage, caused by heat and oxidative stress, which was manifested as an inflammatory process, epithelial tissue sloughing, and mast cell presence. Under unsuitable rearing conditions, these processes may promote cell apoptosis and tissue damage (Sun et al. 2019). Additionally, heat stress and hypercapnia are known to reduce digestive enzyme activity (Pimentel et al. 2015); metabolic and physiological disorders may compromise larval weaning and the transition from the larval to the juvenile stage, which may result in the slight decrease in oxygen consumption found in totoaba reared at 28 °C under current experimental conditions.

Although resting metabolic rate (RMR) considers growth cost, it is advisable to also assess the specific dynamic action (SDA), active metabolic rate (AMR), and maximum metabolic rate (MMR) to evaluate energy expenditure during larval-juvenile fish transition (Chabot et al. 2016; Peck and Moyano 2016). This recommendation is made because totoaba larval stage is characterized by anabolic-catabolic metabolism development (Larios-Soriano et al. 2023), acid digestion (Galaviz et al. 2015), and its transition from stenohaline to euryhaline (Giffard-Mena et al. 2020), which increased weight gain and also oxygen requirements. The change from lecithotrophic to exotrophic feeding promotes and increases in food intake, resulting in an increase in aerobic metabolism that increases ATP production (Van de Pol et al. 2017). This result is reflected in the relationship between k4 and PCA1 (early juveniles aged 24 DAH reared at 26 and 28 °C), where age, weaning, and oxygen consumption were the variables that better explained the observed phenotypic variability. However, the observed trend in O_2_ consumption at 24 DPH suggests lower energy availability to maintain basal metabolism at temperatures below the optimal range. These results suggest that the energetic metabolism presented outside the range of 24–26 °C may be compromised during the larval-juvenile transition, compromising the development and energetic demand of body systems, such as the respiratory, osmoregulatory, immunological, or digestive ones.

## Conclusion

The present results indicate that the optimum culture temperature is close to 26 °C, based on the significant gains in body size in length and weight in totoaba larvae reared at 26 and 28 °C. Temperature-dependent variations at 20 and 24 °C resulted in the formation of two distinct clusters at 24 DPH. This clustering pattern corresponded to reduced mean final body size at 28, 24, and 20 °C compared to the optimal temperature of 26 °C, indicating growth delays of 1, 4, and 5 DPH, respectively. Temperature was also identified, finding it significantly correlated with SGR, which influenced isometric growth at 20 °C and negative allometric growth at 24, 26, and 28 °C. Temperature also had a significant effect on hepatocyte diameter at 20 and 28 °C, together with a significantly higher oxygen consumption than at 26 °C. This result was related to a lower thermal coefficient for routine metabolic rate in the range from 24 to 26 °C and up to 16 DPH.

However, oxygen consumption variability increased significantly from 24 to 24 DPH, which correlated with substantial inflammation of lamellar secondary structures (LS) in the gills. Conversely, at elevated temperature (28 °C), gill morphology was characterized by a significant reduction in lamellae primary (LP) diameter. Therefore, the absence of significant differences in oxygen consumption, combined with the morphological status of gills and hepatocytes at 24 DPH, suggests that metabolic adjustments and phenotypic plasticity facilitated the larval-juvenile transition under suboptimal thermal conditions. The present findings indicate that metabolic adjustments and phenotypic plasticity enabled successful larval-juvenile transition under suboptimal thermal conditions. Finally, the efficacy of early weaning protocols at 26 °C as a mortality reduction strategy warrants further investigation.

## Supplementary Information

Below is the link to the electronic supplementary material.

Supplementary file1 (DOCX 3442 KB)

## Data Availability

No datasets were generated or analysed during the current study.

## References

[CR1] Araujo GS, Silva JWAd, Cotas J, Pereira L (2022) Fish farming techniques: current situation and trends. J Mar Sci Eng 10(11):1598. 10.3390/jmse10111598

[CR2] Ayala A, Muñoz MF, Argüelles S (2014) Lipid peroxidation: production, metabolism, and signaling mechanisms of malondialdehyde and 4-hydroxy-2-nonenal. Oxidative Med Cell Longev 2014:360438. 10.1155/2014/360438 10.1155/2014/360438 PMC4066722 24999379

[CR3] Blagden CS, Currie PD, Ingham PW, Hughes SM (1997) Notochord induction of zebrafish slow muscle mediated by Sonic hedgehog. Genes Dev 11(17):2163–2175. 10.1101/gad.11.17.2163 9303533 10.1101/gad.11.17.2163 PMC275397

[CR4] Boglione C, Gisbert E, Gavaia PE, Witten P, Moren M, Fontagné S, Koumoundouros G (2013) Skeletal anomalies in reared European fish larvae and juveniles. Part 2: main typologies, occurrences and causative factors. Rev Aquaculture 5(2013):121–167. 10.1111/raq.12016

[CR5] Burke M, Grant J, Filgueira R, Swanson A (2022) Oxygenation effects on temperature and dissolved oxygen at a commercial Atlantic salmon farm. Aquacult Eng 99(2022):102287. 10.1016/J.AQUAENG.2022.102287

[CR6] Canada P, Conceição LEC, Mira S, Teodósio R, Fernandes JMO, Barrios C, Engrola S (2017) Dietary protein complexity modulates growth, protein utilisation and the expression of protein digestion-related genes in Senegalese sole larvae. Aquaculture 479:273–284. 10.1016/j.aquaculture.2017

[CR7] Carter JE, Sporre MA, Eytan RI (2022) Larviculture, allometric growth patterns, and gape morphology of the Florida blenny, *Chasmodes saburrae*. Aquaculture 554:738153. 10.1016/J.AQUACULTURE.2022.738153

[CR8] Chabot D, McKenzie DJ, Craig JF (2016) Metabolic rate in fishes: definitions, methods and significance for conservation physiology. J Fish Biol 88(1):1–9. 10.1111/jfb.12873 26768969 10.1111/jfb.12873

[CR9] Clark TD, Sandblom E, Jutfelt F (2013) Aerobic scope measurements of fishes in an era of climate change: respirometry, relevance and recommendations. J Exp Biol 216(15):2771–2782. 10.1242/jeb.084251 23842625 10.1242/jeb.084251

[CR10] Cleveland WS, Grosse E, Shyu WM (2017) Local regression models. Statistical Models S Routledge 2019:309–376

[CR11] Copp GH, Kováč V (1996) When do fish with indirect development become juveniles? Can J Fish Aquat Sci 53(4):746–752. 10.1139/F95-252/ASSET/F95-252.FP.PNG_V03

[CR12] Dahlke FT, Wohlrab S, Butzin M, Pörtner HO (2020) Thermal bottlenecks in the life cycle define climate vulnerability of fish. Science 369(6499):65–70. 10.1126/science.aaz3658 32631888 10.1126/science.aaz3658

[CR13] Downie AT, Illing B, Faria AM, Rummer JL (2020) Swimming performance of marine fish larvae: review of a universal trait under ecological and environmental pressure. Rev Fish Biol Fish 30(1):93–108. 10.1007/S11160-019-09592-W

[CR14] Galaviz MA, García-Ortega A, Gisbert E, López LM, Gasca AG (2012) Expression and activity of trypsin and pepsin during larval development of the spotted rose snapper *Lutjanus guttatus*. Comp Biochem Physiol b: Biochem Mol Biol 161(1):9–16 21925616 10.1016/j.cbpb.2011.09.001

[CR15] Galaviz MA, López LM, García Gasca A, Álvarez González CA, True CD, Gisbert E (2015) Digestive system development and study of acid and alkaline protease digestive capacities using biochemical and molecular approaches in totoaba (*Totoaba macdonaldi*) larvae. Fish Physiol Biochem 41(5):1117–1130. 10.1007/S10695-015-0073-6 25987008 10.1007/s10695-015-0073-6

[CR16] Gamberoni P, Yúfera M, de las Heras V, Siguero I, Gilannejad N, Martínez-Rodríguez G, Navarro-Guillén C (2021) Ontogeny and diurnal patterns of molecular gene expression and activity of digestive enzymes in developing greater amberjack. Aquaculture 534:736330

[CR17] García-Ortega A, Lazo JP (2004) Marine fish larviculture in Mexico: advances and challenges in nutrition and feeding. Avances en Nutrición Acuicola 2004

[CR18] Gibb AC, Swanson BO, Wesp H, Landels C, Liu C (2006) Development of the escape response in teleost fishes: do ontogenetic changes enable improved performance? Physiol Biochem Zool 79(1):7–19. 10.1086/498192 16380924 10.1086/498192

[CR19] Giffard-Mena I, Hernandez-Montiel AH, Perez-Robles J, David-True C (2020) Effects of salinity on survival and plasma osmolarity of *Totoaba macdonaldi* eggs, larvae, and juveniles. J Exp Mar Biol Ecol 526:151339. 10.1016/j.jembe.2020.151339

[CR20] Gisbert E, Ortiz-Delgado JB, Sarasquete C (2008) Nutritional cellular biomarkers in early life stages of fish. Histol Histopathol 23:1525–1539 18830938 10.14670/HH-23.1525

[CR21] Han P, Qiao Y, He J, Wang X (2023) Stress responses to warming in Japanese flounder (*Paralichthys olivaceus*) from different environmental scenarios. Sci Total Environ 897:165341. 10.1016/j.scitotenv.2023.165341 37414161 10.1016/j.scitotenv.2023.165341

[CR22] Häussinger D (1998) Pathogenesis and treatment of chronic hepatic encephalopathy. Digestion 59(2):25–27. 10.1159/000051416 9718415 10.1159/000051416

[CR23] Hernández-Aguilar SB, Zenteno-Savin T, De-Anda-Montañez JA, Méndez-Rodríguez LC (2018) Temporal variation in oxidative stress indicators in liver of totoaba (*Totoaba macdonaldi*) Perciformes: Sciaenidae. J Mar Biol Assoc U K 98(4):833–844. 10.1017/S0025315416001909

[CR24] Hu J, Liu Y, Ma Z, Qin JG (2018) Feeding and development of warm water marine fish larvae in early life. Emerging issues in fish larvae research. Cham: Springer International Publishing 2018:275–296. 10.1007/978-3-319-73244-2_10/COVER

[CR25] Jacob E, Drexel M, Schwerte T, Pelster B (2002) Influence of hypoxia and of hypoxemia on the development of cardiac activity in zebrafish larvae. Am J Physiol-Regulatory Integrative Comparative Physiol 283(4):911–917. 10.1152/ajpregu.00673.2001 10.1152/ajpregu.00673.2001 12228061

[CR26] Jauralde I, Martínez-Llorens S, Tomás A, Ballestrazzi R, Jover M (2011) A proposal for modelling the thermal-unit growth coefficient and feed conversion ratio as functions of feeding rate for gilthead sea bream (Sparus aurata, L.) in summer conditions. Aquaculture Res 44(2):242–253. 10.1111/j.1365-2109.2011.0

[CR27] Jiang D, Wu Y, Huang D, Ren X, Wang Y (2017) Effect of blood glucose level on acute stress response of grass carp *Ctenopharyngodon idella*. Fish Physiol Biochem 43(5):1433–1442 28589315 10.1007/s10695-017-0383-y

[CR28] Jobling M (2003) The thermal growth coefficient (TGC) model of fish growth: a cautionary note. Aquac Res 34(7):581–584

[CR29] Keil G, Cummings E, de Magalhães JP (2015) Being cool: how body temperature influences ageing and longevity. Biogerontology 16:383–397. 10.1007/s10522-015-9571-2 25832892 10.1007/s10522-015-9571-2 PMC4486781

[CR30] Khieokhajonkhet A, Sangphrom S, Aeksiri N, Tatsapong P, Wuthijaree K, Kaneko G (2022) Effects of long-term exposure to high temperature on growth performance, chemical composition, hematological and histological changes, and physiological responses in hybrid catfish [♂*Clarias gariepinus* (Burchell, 1822) ×♀ *C. macrocephalus* (Günther, 1864)]. J Therm Biol 105:103226. 10.1016/J.JTHERBIO.2022.103226 35393060 10.1016/j.jtherbio.2022.103226

[CR31] Khoa TND, Waqalevu V, Honda A, Shiozaki K, Kotani T (2019) Comparative study on early digestive enzyme activity and expression in red sea bream (*Pagrus major*) fed on live feed and micro-diet. Aquaculture 519:734721

[CR32] Killen SS, Costa I, Brown JA, Gamperl AK (2007) Little left in the tank: metabolic scaling in marine teleosts and its implications for aerobic scope. Proc Biol Sci 274(1608):431–438. 10.1098/rspb.2006.3741 17164208 10.1098/rspb.2006.3741 PMC1702384

[CR33] Kjørsvik E, Mangor-Jensen A, Holmefjord I (1990) Egg quality in fishes. Adv Marine Biol 26:71–113. 10.1016/S0065-2881(08)60199-6

[CR34] Koumoundouros G, Ashton C, Xenikoudakis G, Giopanou I, Georgakopoulou E, Stickland N (2009) Ontogenetic differentiation of swimming performance in Gilthead seabream (*Sparus aurata*, Linnaeus 1758) during metamorphosis. J Exp Mar Biol Ecol 370(1–2):75–81. 10.1016/J.JEMBE.2008.12.001

[CR35] Kupren K, Trąbska I, Żarski D, Krejszeff S, Palińska-Żarska K, Kucharczyk D (2014) Early development and allometric growth patterns in burbot Lota lota. Aquacult Int 22(1):29–39. 10.1007/S10499-013-9680-3/FIG.S/6

[CR36] Larios-Soriano E, Díaz F, Re-Araujo AD, López LM, López-Galindo L, True CD, Galaviz MA (2023) Influence of temperature on respiratory metabolism during early development of *Totoaba macdonaldi*. Lat Am J Aquat Res 51(1):109–116. 10.3856/VOL51-ISSUE1-FULLTEXT-2952

[CR37] Lavin CP, Gordó-Vilaseca C, Stephenson F, Shi Z, Costello MJ (2022) Warmer temperature decreases the maximum length of six species of marine fishes, crustacean, and squid in New Zealand. Environ Biol Fishes 105(10):1431–1446. 10.1007/S10641-022-01251-7/FIG.S/6

[CR38] Lema SC, Bock SL, Malley MM, Elkins EA (2019) Warming waters beget smaller fish: evidence for reduced size and altered morphology in a desert fish following anthropogenic temperature change. Biol Lett 15(10):20190518. 10.1098/rsbl.2019.0518 31615375 10.1098/rsbl.2019.0518 PMC6832196

[CR39] López LM, Flores-Ibarra M, Bañuelos-Vargas I, Galaviz MA, True CD (2015) Effect of fishmeal replacement by soy protein concentrate with taurine supplementation on growth performance, hematological and biochemical status, and liver histology of totoaba juveniles (*Totoaba macdonaldi*). Fish Physiol Biochem 41(4):921–936. 10.1007/S10695-015-0058-5 25899616 10.1007/s10695-015-0058-5

[CR40] Luiza T, De Brito CA, Alves Do Nascimento A, Fábio C, Gonçalves S, Antônio M, Assis TL (2020) Assessing the histological changes in fish gills as environmental bioindicators in Paraty and Sepetiba bays in Rio de Janeiro, Brazil. Lat Am J Aquat Res 48(4):590–601. 10.3856/vol48-issue4-fulltext-2351

[CR41] Madeira D, Vinagre C, Diniz MS (2016) Are fish in hot water? Effects of warming on oxidative stress metabolism in the commercial species Sparus aurata. Ecological Indicators 63:324–331. 10.1016/j.ecolind.2015.12.008

[CR42] Maldonado-Othón CA, De La Re-Vega E, Perez-Velazquez M, González-Félix ML (2022) Replacement of fish oil by camelina and black soldier fly larvae oils in diets for juvenile *Totoaba macdonaldi* and their effect on growth, fatty acid profile, and gene expression of pancreatic lipases. Aquaculture 552:737985. 10.1016/J.AQUACULTURE.2022.737985

[CR43] Mata-Sotres JA, Lazo JP, Baron-Sevilla B (2015) Effect of age on weaning success in totoaba (Totoaba macdonaldi) larval culture. Aquaculture 437:292–296. 10.1016/j.aquaculture.2014

[CR44] Osse JWM, Van den Boogaart JGM (2004) Allometric growth in fish larvae: timing and Function. In The development of form and function in fishes and the question of larval adaptation 2004:167–194 https://research.wur.nl/en/publications/allometric-growth-in-fish-larvae-timing-and-function

[CR45] Osse JWM, Van den Boogaart JGM (1995) Fish larvae, development, allometric growth and the aquatic environment. ICES Marine Sci Symposia 201:21–34. 10.17895/ices.pub.19271519

[CR46] Oufiero CE, Whitlow KR (2016) The evolution of phenotypic plasticity in fish swimming. Curr Zool 62(5):475–488. 10.1093/CZ/ZOW084 29491937 10.1093/cz/zow084 PMC5804253

[CR47] Pacorig V, Galeotti M, Beraldo P (2022) Multiparametric semi-quantitative scoring system for the histological evaluation of marine fish larval and juvenile quality. Aquac Rep 26:101285. 10.1016/J.AQREP.2022.101285

[CR48] Peck MA, Moyano M (2016) Measuring respiration rates in marine fish larvae: challenges and advances. J Fish Biol 88(1):173–205. 10.1111/jfb.12810 26768975 10.1111/jfb.12810

[CR49] Pepe-Victoriano R, Miranda L, Ortega A, Merino GE (2021) Descriptive morphology and allometric growth of the larval development of Sarda chiliensis chiliensis (Cuvier, 1832) in a hatchery in northern Chile. Aquac Rep 19:100576. 10.1016/J.AQREP.2020.100576

[CR50] Pimentel MS, Faleiro F, Diniz M, Machado J, Pousão-Ferreira P, Peck MA, Rosa R (2015) Oxidative stress and digestive enzyme activity of flatfish larvae in a changing ocean. PLoS One 10(7):e0134082. 10.1371/JOURNAL.PONE.0134082 26221723 10.1371/journal.pone.0134082 PMC4519323

[CR51] Qin H, Long Z, Huang Z, Ma J, Kong L, Lin Y, Lin H et al (2023) A comparison of the physiological responses to heat stress of two sizes of juvenile spotted seabass (*Lateolabrax maculatus*). Fishes 8(7):340. 10.3390/fishes8070340

[CR52] Réalis-Doyelle E, Pasquet A, De Charleroy D, Fontaine P, Teletchea F (2016) Strong effects of temperature on the early life stages of a cold stenothermal fish species, brown trout (*Salmo trutta* L.). PLoS ONE 11(5):e0155487. 10.1371/JOURNAL.PONE.0155487 27170996 10.1371/journal.pone.0155487 PMC4865038

[CR53] Richard N, Silva TS, Wulff T, Schrama D, Dias JP, Rodrigues PML, Conceição LEC (2016) Nutritional mitigation of winter thermal stress in gilthead seabream: associated metabolic pathways and potential indicators of nutritional state. J Proteomics 142:1–14. 10.1016/J.JPROT.2016.04.037 27126605 10.1016/j.jprot.2016.04.037

[CR54] Ritchie DJ, Friesen CR (2022) Invited review: Thermal effects on oxidative stress in vertebrate ectotherms. Comp Biochem Physiol a: Mol Integr Physiol 263(2022):111082. 10.1016/j.cbpa.2021.111082 34571153 10.1016/j.cbpa.2021.111082

[CR55] Roman MR, Brandt SB, Houde ED, Pierson JJ (2019) Interactive effects of hypoxia and temperature on coastal pelagic zooplankton and fish. Front Mar Sci 6(2019):139. 10.3389/FMARS.2019.00139/BIBTEX

[CR56] Rombough PJ (1999) The gill of fish larvae. Is it primarily a respiratory or an ionoregulatory structure? J Fish Biol 55:186–204. 10.1111/J.1095-8649.1999.TB01055.X

[CR57] Samei EA, Shoman HM, Azab AM (2021) A comparative study on gill histology and ultrastructure of the sea bass (Dicentrarchus labrax) inhabiting brackish, marine, and hyper-saline waters. Egypt J Aquatic Biol Fish 25(6):111–128. 10.21608/ejabf.2021.210812

[CR58] Sánchez-Hernández J, Nunn AD, Adams CE, Amundsen PA (2018) Causes and consequences of ontogenetic dietary shifts: a global synthesis using fish models. Biol Rev 94(2):539–554. 10.1111/BRV.12468 30251433 10.1111/brv.12468

[CR59] Schmidt-Nielsen K (1997) Animal physiology: adaptation and environment, 5th edn. Cambridge University Press, Cambridge

[CR60] Schneider CA, Rasband WS, Eliceiri KW (2012) NIH image to imageJ: 25 years of image analysis. Nat Methods 9(7):671–675. 10.1038/nmeth.2089 10.1038/nmeth.2089 PMC5554542 22930834

[CR61] Shin MG, Ryu YW, Choi YH, Kim SK (2022) Morphological and allometric changes in *Anguilla japonica* larvae. Biology 11(3):407. 10.3390/BIOLOGY11030407 35336781 10.3390/biology11030407 PMC8945780

[CR62] Sprugel DG (1983) Correcting for bias in log-transformed allometric equations. Ecology 64(1):209–210. 10.2307/1937343

[CR63] Srichanun M, Tantikitti C, Utarabhand P, Kortner TM (2013) Gene expression and activity of digestive enzymes during the larval development of Asian seabass (*Lates calcarifer*). Comp Biochem Physiol b: Biochem Mol Biol 165(1):1–9 23458902 10.1016/j.cbpb.2013.02.005

[CR64] Steffensen JF (2005) Respiratory systems and metabolic rates. Fish Physiology 22(2005):203–238. 10.1016/S1546-5098(04)22005-2

[CR65] Sun JL, Zhao LL, Liao L, Tang XH, Cui C, Liu Q, Yang S (2019) Interactive effect of thermal and hypoxia on largemouth bass (Micropterus salmoides) gill and liver: aggravation of oxidative stress, inhibition of immunity and promotion of cell apoptosis. Fish Shellfish Immunol 98(2020):923–936. 10.1016/j.fsi.2019.11.056 31770642 10.1016/j.fsi.2019.11.056

[CR66] Surendran S, Ampili M (2023) Morphological changes and ionic regulations in gills of euryhaline fish during osmoregulation: a review. Uttar Pradesh J Zool 44(18):11–17. 10.56557/upjoz/2023/v44i183599

[CR67] Talamas RE (2001) Temperature effect over the thermal preferendum and metabolism of juvenile Totoaba macdonaldi [Tesis maestría, CICESE]. Repositorio institucional del Centro de Investigación Científica y de Educación Superior de Ensenada https://cicese.repositorioinstitucional.mx/jspui/browse?type=author&value=Eduardo+Talam%C3%A1s+Rohana

[CR68] True CD, Silva LA, Castro N (1997) Acquisition of broodstock *Totoaba macdonaldi*: field handling, decompression, and prophylaxis of an endangered species. Prog Fish-Cult 59(4):246–248. 10.1577/1548-8640(1997)059%3c0246:AOBTMF%3e2.3.CO;2

[CR69] Tseng YC, Hwang PP (2008) Some insights into energy metabolism for osmoregulation in fish. Comp Biochem Physiol C Toxicol Pharmacol 148(4):419–429 10.1016/j.cbpc.2008.04.009 18539088

[CR70] Van de Pol I, Flik G, Gorissen M (2017) Comparative physiology of energy metabolism: fishing for endocrine signals in the early vertebrate pool. Front Endocrinol 8:36. 10.3389/fendo.2017.00036 10.3389/fendo.2017.00036 PMC5332387 28303116

[CR71] Vinagre C, Madeira D, Mendonça V, Dias M, Roma J, Diniz MS (2014) Effect of increasing temperature in the differential activity of oxidative stress biomarkers in various tissues of the Rock goby, *Gobius paganellus*. Mar Environ Res 97:10–14. 10.1016/j.marenvres.2014.01.007 24534436 10.1016/j.marenvres.2014.01.007

[CR72] Volkoff H, Rønnestad I (2020) Effects of temperature on feeding and digestive processes in fish. Temperature 7(4):307–320. 10.1080/23328940.2020.1765950 10.1080/23328940.2020.1765950 PMC7678922 33251280

[CR73] Xu B, Li D, Wei K, Zhu X, Xu J, Ma B (2023) Allometric growth patterns and ontogenetic development during early larval stages of *Schizothorax waltoni* Regan and *Percocypris retrodorslis* in Southwest China. Water 15(4):824. 10.3390/w15040824

[CR74] Xue Y, Zhao J, Deng Y, Wu X, Miao W (2012) Cloning and spatiotemporal expression of pepsinogen and gastric proton pump genes from mandarin fish (*Siniperca chuatsi*) during early ontogeny. Fish Physiol Biochem 39(4):881–893 23184420 10.1007/s10695-012-9748-4

[CR75] Yan W, Qiao Y, He J, Qu J, Liu Y, Zhang Q, Wang X (2022) Molecular mechanism based on histopathology, antioxidant system and transcriptomic profiles in heat stress response in the gills of Japanese flounder. Int J Mol Sci 23(6):3286. 10.3390/ijms23063286 35328705 10.3390/ijms23063286 PMC8955770

[CR76] Yen OEE, Correa Reyes JG, Hernández Rodríguez M (2021) Growth, thermal preference and critical thermal maximum for *Totoaba macdonaldi*: effect of acclimation temperature and inclusion of soybean meal in the diet. Lat Am J Aquat Res 49(2):258–271. 10.3856/VOL49-ISSUE2-FULLTEXT-2563

