# Supplementary material for: The effects of temperature changes on Totoaba macdonaldi larval development, growth, and respiratory rates
Source: Fish Physiol Biochem. 2025 Oct 22;51(6):178. doi: 10.1007/s10695-025-01595-8 (PMC12546414; doi:10.1007/s10695-025-01595-8)
Supplement: Supplementary file 1 — Supplementary file1 (DOCX 3442 KB) [file 10695_2025_1595_MOESM1_ESM.docx]

**Supplementary Material**

Appendix A:

**
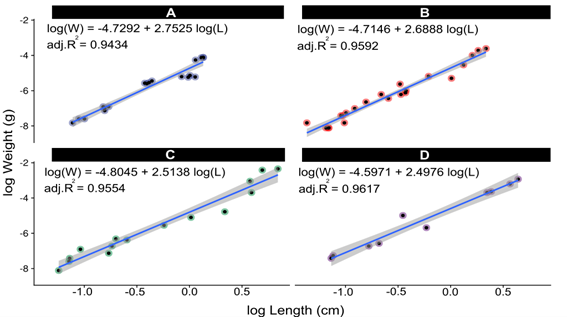
**

**Fig. S1** Fitted linear regression model with transformed weight and length variables. LogW (g): logarithm of weigtn in grams, logL (cm): logarithm of length in cm

**
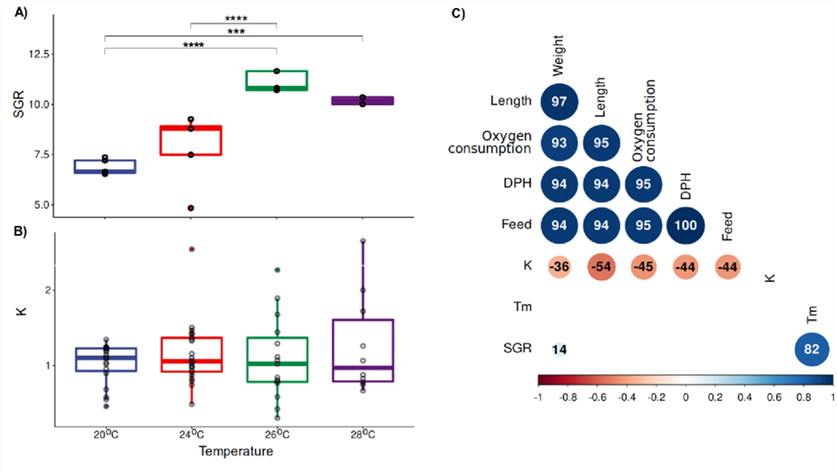
**

**Fig. S2** Statistical analysis of SGR and K condition between different exposure temperatures for totoaba larvae at 24 DPH. a) SGR at different temperatures. b) Condition K at different temperatures. **c)** Spearman correlation coefficient matrix between variables from a confidence interval of 0.95% with significant correlation


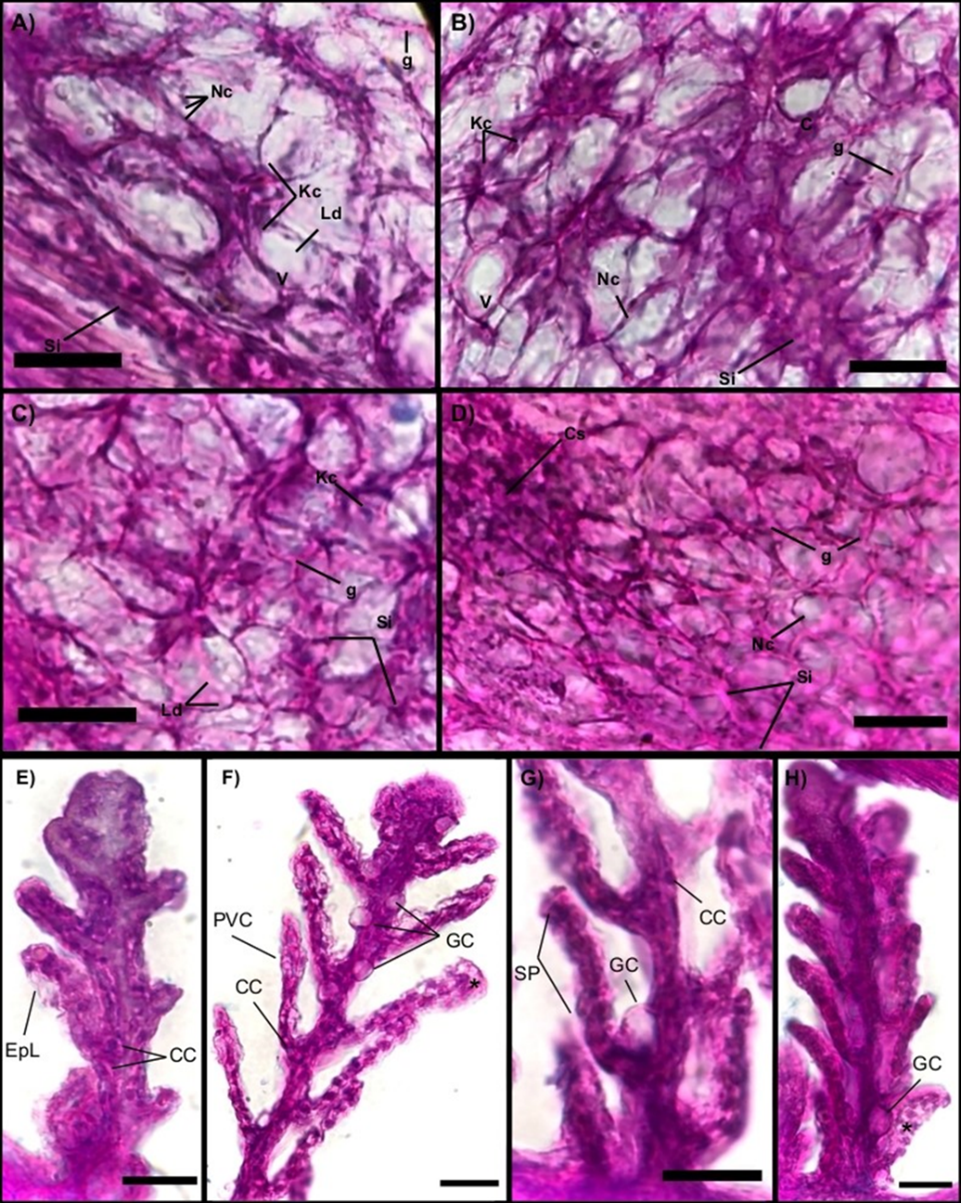


**Fig. S3** Histology of liver and gills of totoaba larvae at 16 DPH. A) and E) Liver and gill filament of totoaba at 20 ºC. B) and F) Liver and gill filament at 24 ºC. C) and G) Liver and gill filament of totoaba at 26 ºC. D) and H) Liver and gill filament at 28 ºC, AB staining, bars = 20 um. PVC: pavement cells, h: hyperplasia, GCH: Globet cell hypertrophy, PF: primary filament, SF: secondary filament, e: erythrocytes, *: lamellae congestion, em: excess mucus, P: pillar cells, SFF: secondary filament fusion, EpL: cell detachment, PFN: primary filament necrosis, CC: chloride cells, Bs: blood sinusoid, Circulus: pyknotic nucleus, V: vacuolization, Si: inflamed sinusoid, Kc: Kupffer cells, IKC: increased Kupffer cells, Rh: hepatocyte rupture, H: hepatocyte, C: central vein, Ld: lipid droplets, g: glycogen, Nc: nucleus compression, Cs: sinusoidal congestion


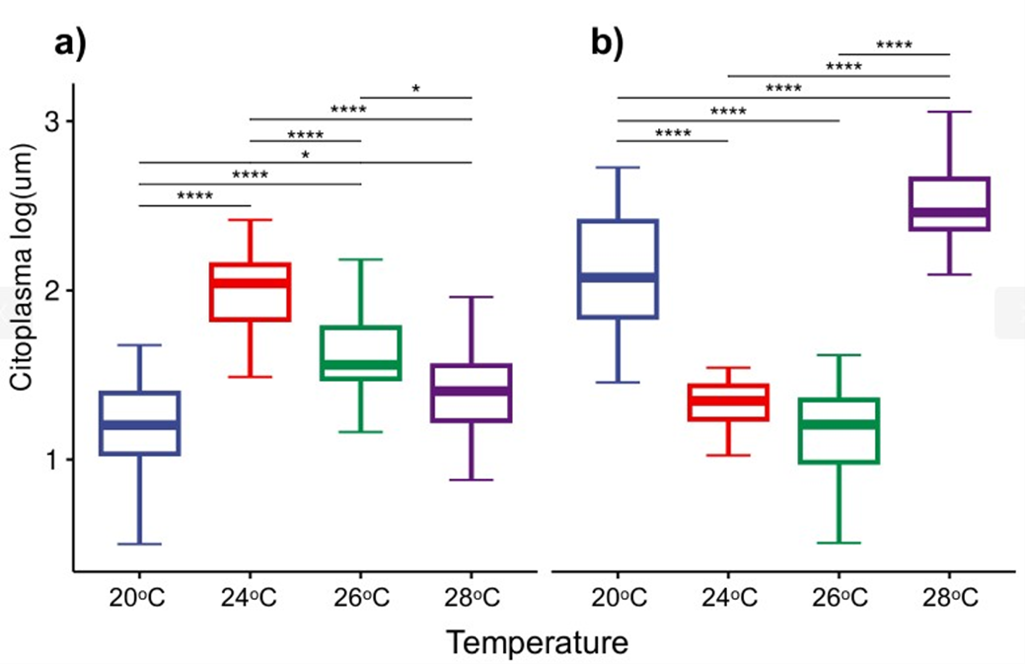


**Fig. S4** Comparison by groups of hepatocyte cytoplasm diameter (um) with Mixed-effects ANOVA and Dunn's post hoc test with Bonferroni adjustment between dhp 16 and 24. a**)** Post-hoc analysis for DPH 16 (p. adj <0.05). **b)** Post-hoc analysis for DPH 24 (p. adj <0.05). Significant differences were observed (p<0.05, η2 = 0.68) and higher effect size related to culture temperature (p<0.05, η2 = 0.33). While by treatment D had the largest effect size (p. adj <0.05, η2 = 0.82)
